# Update on Some Aspects of Neonatal Thyroid Disease

**DOI:** 10.4274/jcrpe.v2i3.95

**Published:** 2010-08-01

**Authors:** Tamar Simpser, Robert Rapaport

**Affiliations:** 1 Mount Sinai School of Medicine, Pediatric Endocrinology, New York, NY, USA; +1 212 2416936tamar.simpser@gmail.comMount Sinai School of Medicine, Pediatric Endocrinology, New York, NY, USA

**Keywords:** congenital hypothyroidism, thyroxine

## Abstract

This article explores the basic development and pathophysiology of the thyroid gland. New factors in the normal development of the thyroid in the neonate are mentioned. The incidence of congenital hypothyroidism continues to increase. We describe congenital hypothyroidism, its possible etiologies, treatment and outcomes. We explore hypothyroxinanemia in pre−term neonates and the risk/benefit of prophylactic thyroid hormone replacement. We discuss the late rise of thyrotropin (TSH) in ill infants and those with very low birth weight. Ill infants or those born premature should have their thyroid function tests routinely monitored. On the occasion of borderline thyroid function test results, TRH testing can be useful in identifying those infants with either persistent or transient hypothyroidism. TRH testing is also helpful in identifying those patients with secondary hypothyroidism. With the early identification and prompt and proper treatment, neonates with congenital hypothyroidism, transient or persistent, should have positive long−term outcomes.

**Conflict of interest:**None declared.

## INTRODUCTION

Goiter was first described between the years 2838−2698 B.C.E in the book Pen−Ts’ao Tsing (A Treatise on Herbs and Roots). The supposed author, emperor Shen−Nung, recommended the seaweed Sargassum as an effective remedy for goiter (1). It was not until the 1^st^ century B.C.E. that the writings of Roman authors Vitruvius, Pliny the Elder and Juvenal, made reference to endemic goiter in a region of the Alps. Although already recognized as a medical condition, congenital hypothyroidism was not mentioned in medical texts until year 1300 by Arnaldus de Villanova (goitres in Lucca) and Lanfrancus (goitres in Lombardy) (2). There have been many advances in the study of congenital hypothyroidism since then, most notably in the past forty years.

Normal thyroid function is essential for the growth and neurodevelopment of infants and young children. Abnormalities of thyroid gland development, migration and function can all lead to congenital hypothyroidism. Recent reviews have included defects in the sodium−iodide transporter and the thyrotropin (TSH) receptor, as well as the transcription factors (TFs) PAX−8, TTF1, TTF2 and others, all of which may be associated with abnormalities in thyroid function.

The development of the normal fetal−neonatal thyroid system can be categorized in three phases. The first one begins with thyroid and pituitary embryogenesis occurring up to the 10^th^−12^th^ weeks of gestation. The histologic and functional maturation of the hypothalamus and of the pituitary portal vascular systems begins at 4th−5th gestational weeks and continues through gestational weeks 30−35. The third and final phase of fetal thyroid development is the maturation of the hypothalamic−pituitary−thyroid axis beginning at mid−gestation and continuing through to approximately 4 weeks postnatally. One can easily infer that infants born before term may have disruption in the normal maturation of the fetal hypothalamic−pituitary−thyroid axis leading to abnormal thyroid function.

Genetic defects in transcription factors have been described in relatively few patients. There is great variability between genotype and phenotype in affected individuals. For example, the same defect in PAX 8 may result in anywhere from a normal to an absent thyroid gland and from euthyroidism to severe hypothyroidism (3, 4, 5, 6, 7, 8) (Table 1). Some of the TFs involved in thyroid gland development are also involved in the development of other tissues, such as the kidneys and lungs. There is an increased odds ratio of 13.2 for having renal and urinary tract abnormalities in children with congenital hypothyroidism versus children without congenital hypothyroidism (9). For further details of thyroid gland development, several reviews are recommended (10, 11, 12).

Thyroid function of the neonate can be affected by the mother’s thyroid status by way of placental transfer. While TSH is not transferred from the mother, small amounts of thyroxine (T4) and triiodothyronine (T3) do cross the placental barrier. Thyroid antibodies, both stimulatory and inhibitory, as well as anti−thyroid medication easily cross the placenta and are transferred from the mother to the fetus. For example, the thyroid stimulating immunoglobulins (TSI) from a mother with Graves disease will cross the placental barrier and can result in transient hyperthyroidism in the neonate. If this same mother is on treatment with thioamides, which also cross the placental barrier, the neonate can develop transient hypothyroidism. The possible impact of the mother’s thyroid status could present a difficult challenge to the physician in diagnosing thyroid abnormalities in the neonate. We have recommended measurements of TSI in such newborns as helpful in assessing the likelihood of developing symptomatic hyperthyroidism (13).

Results of thyroid function tests (TFTs) vary with age. There is a rise in TSH, T3 and T4 immediately after birth. These measures of thyroid function will gradually decrease by 3−4 days of age. It is important to note that specimens obtained on the first day of life might have a high TSH level with high levels of T3 and T4. TFTs in babies born prior to term need to be interpreted with careful regard for age and gestational age. T4 and T3 are especially affected. TSH levels do not vary as greatly during the newborn period.

If neonates with congenital hypothyroidism are left untreated, they will develop severe symptoms of hypothyroidism, such as developmental and growth retardation. In order to avoid potential damage to the developing brain caused by congenital hypothyroidism, newborn screening was instituted in Quebec, Canada in 1974. The frequency of congenital hypothyroidism was found to be 1:7000 and the screening program was continued (14). With the expansion of newborn screening programs, the frequency of congenital hypothyroidism continues to increase in the United States (US) and worldwide and is now thought to be around 1:3000−4000. In New York State alone, between 1978 and 2005, the incidence of congenital hypothyroidism has increased by 138% to approximately 1:1415 births. In the US (excluding New York State data), with nearly 58 million infants screened between 1987 and 2002, the incidence has increased 73% (15). The largest proportion of newborn screening programs worldwide and in the US use a primary T4 screen with TSH measured to confirm the lowest T4 results. Other countries and fewer US states use a primary TSH screen alone. In the US, a limited number of states perform both a primary T4 and TSH screen at the initial screening (16, 17).

It is important to note that two−thirds of infants diagnosed with congenital hypothyroidism had neither signs nor symptoms of hypothyroidism. Transient hypothyroxinemia is common in neonates born prematurely. Thyroxine−binding globulin (TBG) deficiencies occur in 1:9000, while TSH and/or TRH deficiencies occur in less than 1:20000−100000. A study in the Netherlands that screened 385000 infants over a two−year period found that congenital hypothyroidism of central origin occurs more often than previously thought. They reported an incidence of 1:20000, representing 13.5% of all cases of permanent congenital hypothyroidism. Over three quarters of the permanent cases of congenital central hypothyroidism had multiple pituitary hormone deficiency and half had pituitary malformations (18).

Treatment for congenital hypothyroidism is thyroid hormone replacement. The current recommended dose is 10−15 μg/kg/day of thyroxine, to be given orally as crushed tablets. Treatment should be instituted as early as possible, in order to optimize cognitive and physical development. Most children do well with treatment, and higher initial thyroxine dosage combined with shorter time to normalization contribute to improved developmental outcome (19, 20).

Some suggest that the dose of thyroid hormone required may be directly related to the etiology of the hypothyroidism. Infants with athyreosis require a higher dose than those with dysgenesis, who require a higher dose than those with dyshormonogenesis (21). Contrary to previous beliefs, congenital hypothyroidism, even due to thyroid dysgenesis, may not be a random occurrence. In a 2001 review of 19 years of newborn screening in France, Castanet et al (22) found that familial cases were reported in a high proportion of children with thyroid dysgenesis, suggesting the possibility of a genetic component. A further review in 2010 by Castanet et al (23) estimates the prevalence of familial cases of thyroid dysgenesis at 2%.

Treatment with thyroid hormone has generally been quite safe and effective. In young adults diagnosed through the national neonatal screening program in the Netherlands, it was found that in neonates with severe congenital hypothyroidism,cognitive and motor defects persist through young adult life. The median age of start of treatment was 28 days after birth and was not an important factor in determining long−term cognitive and motor outcome. The study concludes that the severity of congenital hypothyroidism is inversely correlated with IQ and motor scores (24). Impaired diastolic function and exercise capacity in young adults treated with levothyroxine for congenital hypothyroidism has also been reported. Compared with controls, hypothyroid patients exhibited a higher frequency of left ventricular diastolic dysfunction, impaired exercise capacity, and increased intima−media thickness. Close monitoring of thyroid hormone replacement therapy can prevent cardiovascular abnormalities related to episodes of subclinical hypothyroidism and hyperthyroidism (25).

In neonates with an initial abnormal screen accompanied by normal serum T3 and T4 levels and only mildly elevated TSH, one is faced with a conundrum of mild abnormalities. Based on a 1993 study, we recommended thyroid releasing hormone (TRH) testing for children who had mildly elevated baseline TSH values in order to identify those infants in whom thyroid hormone replacement may be needed, even if transiently (26). This approach was substantiated in an article by Daliva et al (27) who showed that 13 of 14 children with borderline TSH levels at screening had thyroid function anomalies that persisted at three years of age. Of those 13 children, three underwent TRH testing, with two exhibiting hyper−responsiveness (>35mU/L) (27, 28). Recent studies have shown that certain genetic defects can result in either mild or transient hypothyroidism (29, 30, 31) (Table 2). Therefore, TRH testing should be recommended to identify cases of mild congenital hypothyroidism. In addition, TRH testing has a role in diagnosing central hypothyroidism and isolated TSH deficiency (32).

A common finding in preterm newborns is neonatal hypothyroxinemia. Neonatal hypothyroxinemia has been associated with cerebral palsy, mortality, morbidity, and intraventricular hemorrhage (33, 34). Attempts have been made to replace T4 in premature infants with limited demonstrable results (35, 36, 37). In 2001, we recommended that hypothyroxinemia of the preterm infant should not be routinely treated in Neonatal Intensive Care Units, unless aspart of an ongoing study (38). In 2007, a Chochrane Database Systematic Review also did not support the use of prophylactic thyroid hormones in preterm infants to reduce neonatal mortality, neonatal morbidity or improve neurodevelopmental outcomes (39). Prospective studies of thyroid hormone supplementation in premature neonates are underway (40).

Late rise in TSH (LRT) is defined as an elevated TSH level following a normal TSH on the initial newborn screen, with either a normal or a low T4. LRT can occur in premature or ill infants and babies with very low birth weight. We have recommended routine monitoring of TFTs in ill newborns (41). Possible causes of LRT are developmental delays in the maturation of the hypothalamic−pituitary−thyroid axis, exposure to medications and antiseptics, and recovery from sick euthyroid syndrome. A one−year study found 14 infants who had late rise in TSH. Nearly half the cases (n=6) resolved spontaneously, while the remaining 8 infants required thyroid hormone replacement. Of these 14 infants, 79% were premature and 7 had very low birth weight (VLBW). Ten of these infants underwent surgery and of those, 6 had elevated urine iodine. We concluded that LRT in ill newborns may be a common finding and not necessarily associated with birth weight (42).

Abnormalities of thyroid function tests are common in newborns. Particular attention should be paid to recognizing specific disorders of the thyroid gland. It is imperative that any thyroid function abnormalities be identified and treated as early as possible to ensure the best neurodevelopmental and growth outcomes. Genetic studies have already helped to identify the etiology of congenital hypothyroidism in some infants. Expanded genetic screening is likely to be more available in the near future.

**Table 1:**
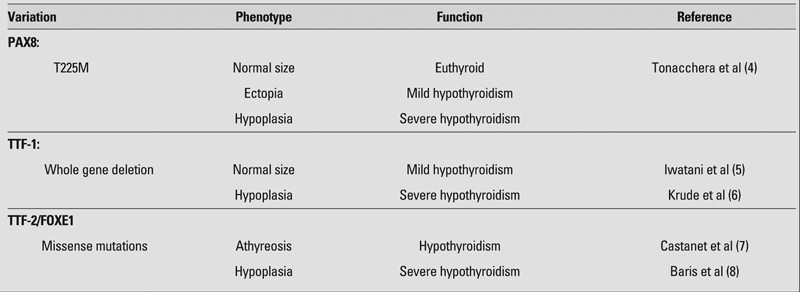
Genetic mutations and variant phenotypes

**Table 2:**
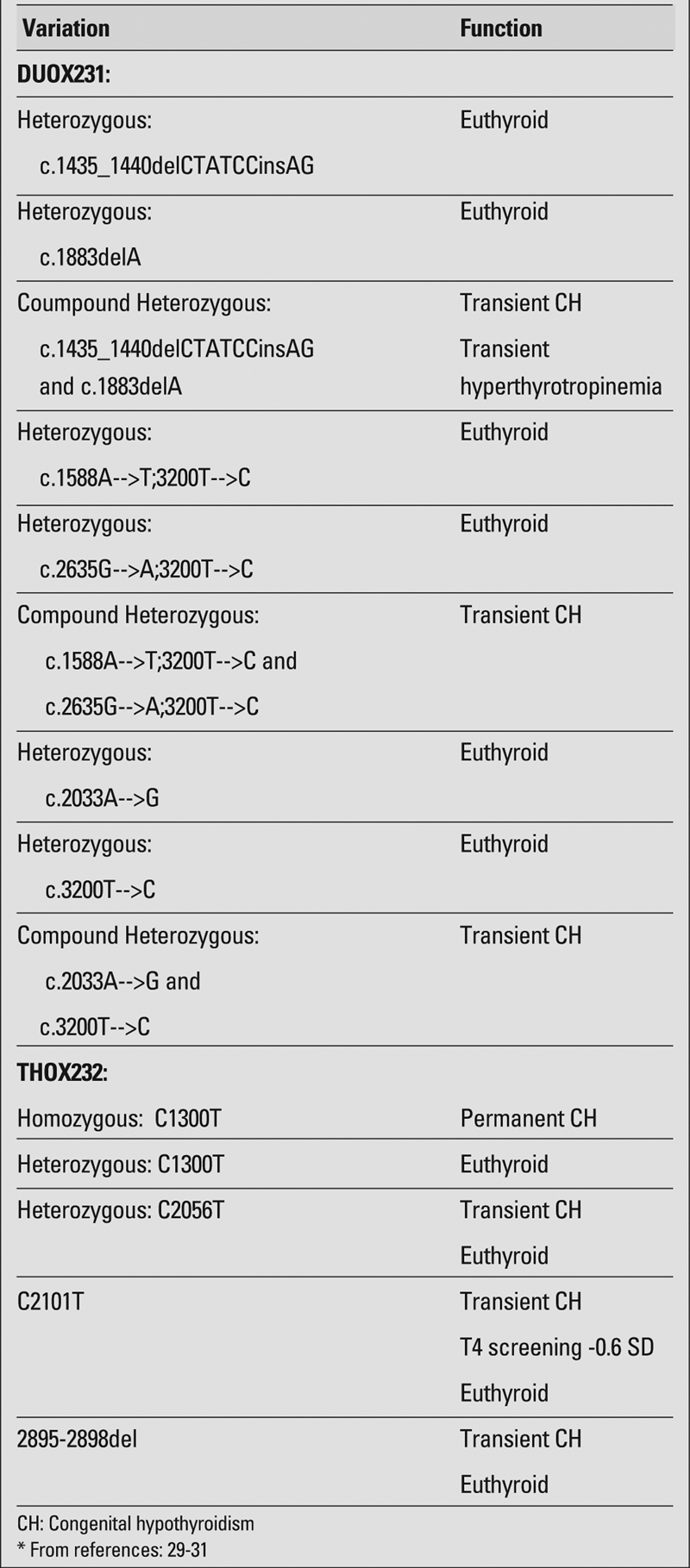
Genetic mutations and thyroid function*
